# Dental education during the pandemic – cross-sectional lecturer-side evaluation for the use of digital teaching concepts

**DOI:** 10.12688/f1000research.122274.1

**Published:** 2022-07-11

**Authors:** Ephraim Nold, Vivienne Demeter, Kurt-Jürgen Erdelt, Daniel Edelhoff, Anja Liebermann

**Affiliations:** 1 Department of Prosthetic Dentistry, Hospital of Ludwig Maximilian University of Munich, Munich, Bavaria, 80336, Germany; 2 Faculty of Medicine and University Hospital Cologne, University of Cologne, Polyclinic of Prosthetic Dentistry, Cologne, North Rhine Westphalia, 50931, Germany

**Keywords:** pandemic, digital teaching concept, lecturer-side evaluation, questionnaire, lecture

## Abstract

**Background:** The COVID-19 pandemic resulted in significant restrictions on dental teaching. The aim of this investigation was to evaluate the attitudes of faculty members towards digital teaching formats and the effort creating digital lectures. We hypothesized that on the lecturer side there is no difference between the various digital teaching concepts in terms of workload and effort and that there is no increase in workload and effort when switching to digital teaching concepts.

**Methods:** All German dental faculties were invited to the online survey by an anonymous voluntary questionnaire from January to April 2021. The questionnaire consisted of 27 questions that could be answered with a visual analog scale, free text answers, or with fixed answer options. Data was analyzed using the Kolmogorov-Smirnov test and an exploratory data analysis (α=0.05).

**Results:** Before the pandemic, 24.8% of the participating lecturers were using digital teaching and 64.4% had no previous experience. After the outbreak of the pandemic 100% of the dental teaching was initially held online. More than 80% of the lecturers stated that they offer online lectures (86.1%), online seminars (81.2%), and/or online bedside teaching (33.7%). 88.1% see face-to-face teaching as the preferred teaching format. The lecturers also see the greatest opportunities for interaction in the area of analog teaching and significantly worse in synchronous and asynchronous digital teaching. In the course of the pandemic, respondents' attitudes towards online teaching improved in the median of 24.0 to a median of 50.0.

**Conclusions:** Faculty members have positively changed their attitudes towards online teaching formats over the course of the pandemic. Although they see the greatest learning success in conventional face-to-face teaching formats and the creation of digital lectures is associated with a higher effort, they want more online lessons in the future.

## Introduction

The use of digital media in university teaching, also called e-learning, has been a popular form of teaching and learning for more than 20 years and has experienced a significant boom since the 2000s. ^1^ In particular, students appreciate the flexible online access to the digital teaching tools as well as the individual adaptability regarding different learning speeds. ^2^ Investigations show, however, that face-to-face teaching, especially personal feedback from the faculty, cannot be completely replaced. ^2^^–^^5^

The COVID-19 pandemic with its worldwide restrictions in the field of dental education and the need to discontinue face-to-face teaching in many places showed ^6^ that the implementation of digital learning as well as teaching support in dental training still has deficiencies. ^7^ However, the call for digital teaching concepts, especially web-based offers, became louder. ^8^

The effectiveness of digital teaching concepts has already been proven by numerous authors. ^9^^–^^11^ Particularly in the area of blended learning, an effectiveness that was not lower than in the case of face-to-face teaching was reported. ^3^ The acceptance of distance learning by students has also been examined in numerous investigations and positive results were reported almost without exception. ^10^^,^^12^^–^^17^

Although the use of digital teaching concepts was extensively described by the students, investigations that examined the topic on the part of the lecturers can hardly be found. ^18^ In addition to the effectiveness of the teaching method mentioned and the acceptance by the students, the question of the creation effort for digital teaching units, the acceptance on the lecturer’s side and their ability to use digital media is a decisive factor for the increased use of digital teaching concepts within the dental curriculum.

Although Schlenz *et al.,* ^18^ reported in an investigation about the acceptance of online teaching of a high level of acceptance by both students and lecturers, but there is little to be found in the literature on the question of creation effort for online teaching concepts. Zitzmann *et al.,* also assumed in their systematic review of the use of digital teaching concepts in dental education that, despite the considerable effort involved in creating digital courses, a long-term relief of the teaching effort can be expected, ^2^ and August *et al.,* ^19^ proposed that lecturers to work together across universities due to the high time required to create digital content in order to minimize the overall effort, whereas a quantification of the creation effort was not mentioned.

The technological progress of the past 10 years has created countless new options in the field of teaching and enabled to practice digital teaching in different ways. ^20^ Concerning asynchronous teaching formats, the outstanding strength is that they can be consumed flexibly in time and place and thus made available to a theoretically unlimited audience over a longer period of time. ^21^^,^^22^ Synchronous formats offer, also without being tied to a specific location, the advantage of direct communication between lecturer and student.

Therefore, the aim of this cross-sectional investigation is to analyze the lecturer-sided acceptance and teaching effort during and after the switch to digital teaching during the pandemic as well as to examine the differences between the various digital teaching concepts as survey-based research. In addition, an insight into the nationwide implementation of dental online teaching is given.

The hypothesis states that on the lecturer side 1. there is no difference between the various digital teaching concepts in terms of workload and effort, and 2. there is no increase in workload and effort when switching to digital teaching concepts.

## Methods

### Ethical statement

A declaration of no objection was approved by the ethics committee of the Medical School (Project KB 20/036). Written informed consent was obtained from participants prior to inclusion in the study.

### Study design

All dental schools, like all other universities were forced to replace their conventional lectures in the auditorium during the pandemic period, by different digital teaching concepts. These digital concepts were performed for lectures, seminars, and bedside teaching, whereby the exact use of the various online concepts can now be assumed to be known. Most digital teaching concepts used were:

1. asynchronous (e.g., prerecorded PowerPoint presentations with audio explanations),
2. synchronous using livestreams, and
3. synchronous using conference systems (e.g., Zoom, Big Blue Button, Jitsi as examples).

This cross-sectional study was a survey-based research by an online questionnaire among dental lecturers from different dental schools at German university hospitals, including various departments responsible for dental teaching.

### Participant recruitment

All German dental schools were invited for the online survey by sending a link for an anonymous online questionnaire by e-mail. The link was sent directly to the heads of the departments of prosthodontics in all German universities and they forwarded the link to their employees within the department. There were no specific exclusion or inclusion criteria. Participants had to be lecturers at dental schools. The questionnaire time frame for recruitment was from January to April 2021.

### Questionnaire development

**Figure 1.**
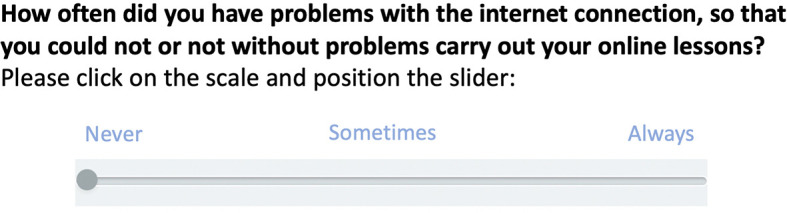
Example of scroll bar question.

The questionnaire was generated using an online survey platform (Questionstar, Hannover, Germany) and consisted of 27 questions (Questions: Q) in the German language. A total of seven questions could be answered using a visual analog scale (VAS), three free text answers, and 17 with fixed-answer options ( Table 1). ^32^ The VAS answers were marked by the students with a scroll bar on a line, which reflected the range from 0% to 100% ( Figure 1). The questions with fixed-answer options were marked just with a click. Some questions were specifically asked in case the different teaching formats were held. Therefore, it was possible that some questions were not answered by the lecturers if the respective teaching format was not held. Before the questionnaire was sent out, it was validated internally by five highly experienced staff members of the Department of Prosthodontics in Munich. All of these staff members and researchers included are active as lecturers at the University of Munich and have more than five years of experience. The questionnaire was discussed in the research group and checked by the study directors to establish content validity. After validation, a short cut was created from Q7 to Q10-12, 17-19, whereas Q20, 22 and Q6 was swapped with Q7. The data was collected from the platform Questionstar (Questionstar, Hannover, Germany) and made available as an Excel file. Data analysis was performed with the help of a research engineer with a high level of statistic expertise.

**Table 1.** Detailed questionnaire used.

Question No.	Question	Answer possibility
**1**	Please specify your gender.	a) Male b) Female c) Diverse
**2**	What is your age?	a) <30 years b) 30-40 years c) 41-50 years d) 51-60 years e) >60 years
**3**	At which German university are you employed?	(Free field to fill in)
**4**	What position do you currently have at your university hospital?	a) Guest lecturer b) Research assistant c) Functional senior physician, senior physician, managing senior physician, director d) None of the above positions
**5**	How much of your overall work is teaching?	a) <25% b) 25-50% c) 51-75% d) >75%
**6**	How many years have you been involved in teaching?	a) <2 years b) 2-5 years c) 6-10 years d) >10 years
**7**	What type of course do you teach?	a) Lectures b) Seminars c) Bedside teaching d) None
**8**	Please indicate in what ways you were actively involved in online teaching prior to the COVID-19 pandemic.	a) I have already taught online courses. b) I have participated in one or more training events on the topic of online teaching. c) I have studied online teaching on my own. d) I have no experience in online teaching before the pandemic.
**9**	Where do you most often hold your online lectures from?	a) Home office/home b) Own office in the university/clinic c) Teaching rooms (lecture halls, conference rooms) d) No answer
**10**	What teaching format did you use to implement the online **lectures**?	a) Synchronous formats such as live online lectures (e.g. WebEx, ZOOM, BigBlueButton, Jitsi). b) Synchronous formats such as broadcasts of lectures held conventionally in the lecture hall and streamed live. c) Asynchronous formats such as PowerPoint presentations set to music that are freely available for self-study via online platforms such as Moodle. d) Other formats: (Free field to fill in)
**11**	What teaching format did you use to implement the online **seminars**?	a) Synchronous formats such as live online lectures (e.g. WebEx, ZOOM, BigBlueButton, Jitsi). b) Synchronous formats such as broadcasts of lectures held conventionally in the lecture hall and streamed live. c) Asynchronous formats such as PowerPoint presentations set to music that are freely available for self-study via online platforms such as Moodle. d) Other formats: (Free field to fill in)
**12**	What teaching format did you use to implement the online bedside-teaching?	a) Synchronous formats such as live online lectures (e.g. WebEx, ZOOM, BigBlueButton, Jitsi). b) Synchronous formats such as broadcasts of lectures held conventionally in the lecture hall and streamed live. c) Asynchronous formats such as PowerPoint presentations set to music that are freely available for self-study via online platforms such as Moodle. d) Other formats: (Free field to fill in).
**13**	How often did you have problems with the internet connection, so that you could not or not without problems carry out your online lessons?	VAS (visual analog scale) range: 0% as never – 100% as always.
**14**	How often did you have problems using the online teaching format, so that you could not or could not carry out your teaching without problems?	VAS (visual analog scale) range: 0% as never – 100% as always.
**15**	Were you directed which online teaching format to use?	a) Yes b) No
**16**	What teaching formats do you prefer to work with?	a) Conventional teaching formats b) Online teaching formats
**17**	Which online teaching format do you personally like best for **lectures** and would recommend to others?	a) Zoom/WebEx/Jitsi/BigBlueButton b) Live broadcast of conventionally held lectures c) Prerecorded lectures with audio (e.g. PowerPoint presentation)
**18**	Which online teaching format do you personally like best for **seminars** and would recommend to others?	a) Zoom/WebEx/Jitsi/BigBlueButton b) Live broadcast of conventionally held seminars c) Prerecorded seminars with audio (e.g. PowerPoint presentation)
**19**	Which online teaching format do you personally like best for **bedside-teaching** and would recommend to others?	a) Zoom/WebEx/Jitsi/BigBlueButton b) Live broadcast of conventionally held bedside-teaching c) Prerecorded bedside-teaching with audio (e.g. PowerPoint presentation)
**20A**	How high do you generally rate the learning success of courses for students?	a) Analog teaching event (Low to high) b) Synchronous formats such as live online or livestreams of lectures, seminars, etc. (Low to high) c) Asynchronous formats like PowerPoint presentations with audio (Low to high)
**20B**	In general, how would you rate the opportunities for interaction with students during classes?	a) Analog teaching event (Highly negative to highly positive) b) Synchronous formats such as live online or livestreams of lectures, seminars, etc. (Highly negative to highly positive) c) Asynchronous formats like PowerPoint presentations with audio (Highly negative to highly positive)
**21A**	What is your attitude towards online teaching?	a) Before the switch to online-only teaching (Highly negative to highly positive) b) At the present time (Highly negative to highly positive)
**21B**	How much theoretical knowledge do you have about online teaching?	a) Before the switch to online-only teaching (Low to high) b) At the present time (Low to high)
**21C**	How high do you rate your own competence regarding the implementation of an online course?	a) Before the switch to online-only teaching (Low to high) b) At the present time (Low to high)
**21D**	How much of a personal workload do you think your teaching job creates for you?	a) Before the switch to online-only teaching (Low to high) b) At the present time (Low to high)
**22A**	What was/is your time commitment for creating a lecture?	a) Analog lecture (Low to high) b) Digital lecture (Low to high)
**22B**	What was/is your time commitment for delivering a lecture?	a) Analog lecture (Low to high) b) Digital lecture (Low to high)
**22C**	What was/is your time commitment to update an existing lecture?	a) Analog lecture (Low to high) b) Digital lecture (Low to high)
**23A**	What was/is your time commitment for creating a seminar?	a) Analog seminar (Low to high) b) Digital seminar (Low to high)
**23B**	What was/is your time commitment for delivering seminar?	a) Analog seminar (Low to high) b) Digital seminar (Low to high)
**23C**	What was/is your time commitment to update an existing seminar?	a) Analog seminar (Low to high) b) Digital seminar (Low to high)
**24A**	What was/is your time commitment for creating a bedside-teaching?	a) Analog bedside-teaching (Low to high) b) Digital bedside-teaching (Low to high)
**24B**	What was/is your time commitment for delivering bedside-teaching?	a) Analog bedside-teaching (Low to high) b) Digital bedside-teaching (Low to high)
**24C**	What was/is your time commitment to update an existing bedside-teaching?	a) Analog bedside-teaching (Low to high) b) Digital bedside-teaching (Low to high)
**25**	Please indicate the % of your courses that you offered online prior to the pandemic.	(Free field to fill in)
**26**	Please indicate the % of your courses that you would like to offer online in the future.	(Free field to fill in)
**27**	Do you think that in the future (regardless of the COVID-19 pandemic) online teaching should be more firmly anchored in dental education in principle?	a) Yes b) No

### Data analysis

The questionnaires were examined with the statistical program SPSS 26 (IBM, New York, NY, USA) with a significance level of p=0.05. Normality of data distribution was analyzed using the Kolmogorov-Smirnov test and an exploratory data analysis. The median values of the questions and the range of deviation of the interquartile range (IQR) were used due to non-parametric analysis. In addition, the Friedmann and Wilcoxon test was performed to compare the results and respective answers.

## Results

A total of 101 lecturers (46 women, 55 men) participated in the survey with a drop-out of 17 lecturers with incomplete questionnaires (drop-out rate: 14%). All results (100%) showed a deviation from the normal distribution and were consequently evaluated non-parametrically.

**Figure 2.**
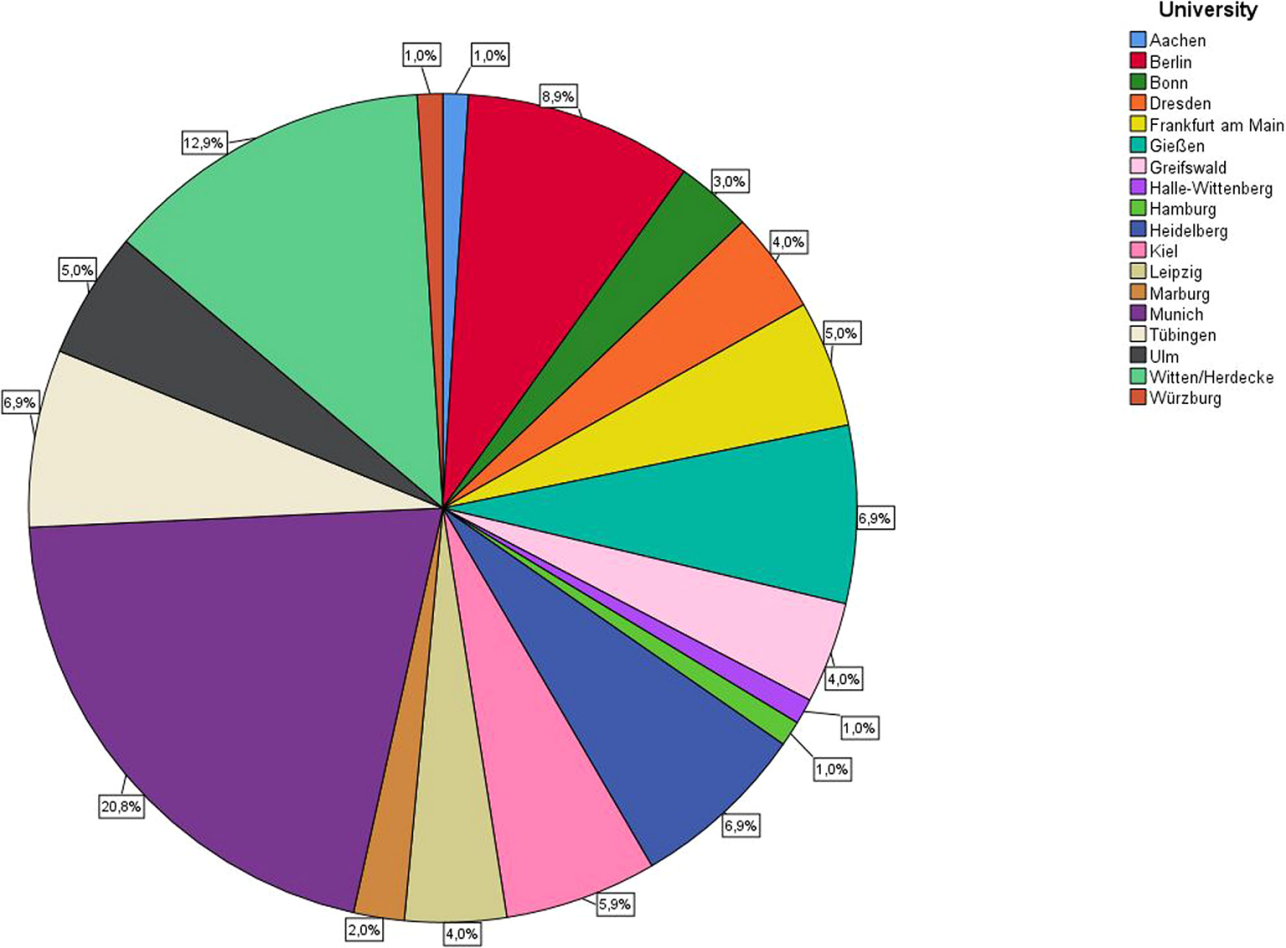
Overview of universities and distribution of participation in the online survey.

Figure 2 shows the distribution of the lecturers at the different universities. A total of 13.9% were under 30 years of age, 35.6% between 30 and 40 years of age, 16.8% between 41 and 50 years of age, 15.8% between 51 and 60 years of age, and 17.8% over 60 years of age.

Among the participating faculty, 3% reported their position within the university as visiting lecturer, 37.6% as research associate, 55.4% as functional/senior physician/chief senior physician or director, and 4.0% with no information.

Regarding the percentage of teaching in the total activity, 13.9% of the lecturers indicated a percentage value of less than 25%, 51.5% indicated a percentage value between 25 and 50%, 28.7% indicated a value of 51 to 75%, and 5.9% of the lecturers even indicated a teaching percentage of more than 75%.

When asked about years of previous teaching experience, 11.9% lecturers reported having less than two years of teaching experience. 18.8% of lecturers indicated teaching experience between two and five years, 17.8% between six and 10 years, and 51.5% with over half of lecturers indicated teaching experience of over 10 years.

Among lecturers, online lectures were presented from the university’s own office by 67.3%, from teaching spaces such as lecture halls or conference rooms by 15.8%, from home office by 13.9%, and 3.0% gave no response. Overall, 86.1% of all lecturers held online lectures, 81.2% online seminars, and 33.7% online bedside teaching since the pandemic initially forced to teach 100% online. Among these, 24.8% of the lecturers reported that they had held online events prior to the pandemic and 25.7% had attended training events on online teaching. 36.6% of the participating lecturers had also dealt with online teaching in self-study before the pandemic. However, 64.4% of the lecturers stated that they had no experience with online teaching prior to pandemic.

**Figure 3.**
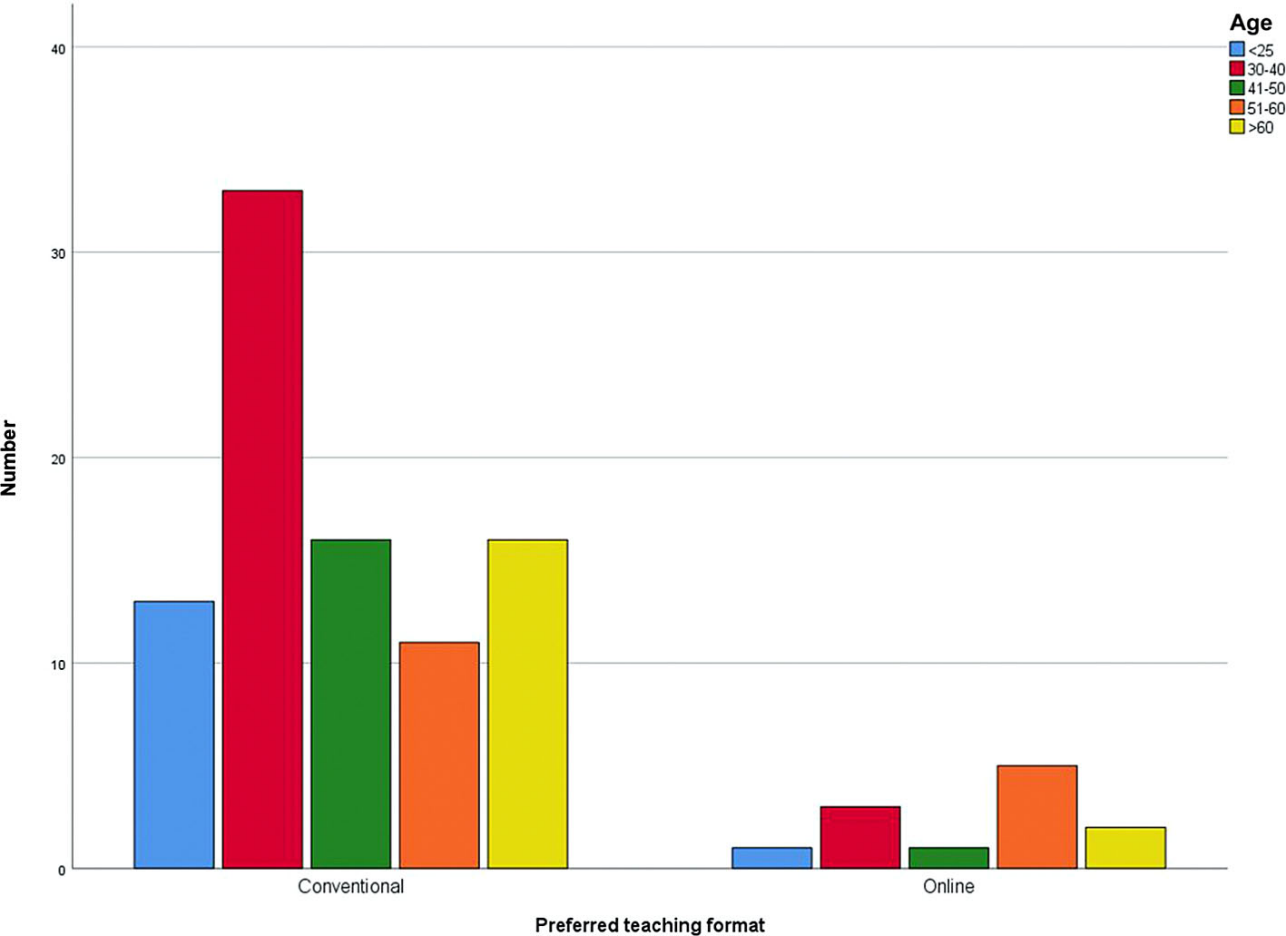
Preferred teaching format for different age groups, separately.

62.4% of the lecturers stated that the university had given them the online teaching format. In contrast, 37.6% of the lecturers were able to determine the online teaching format themselves. 88.1% of the lecturers prefer to work with the conventional teaching format, i.e., face-to-face teaching, and 11.9% with online teaching. The desired teaching format, depending on the age group is shown in Figure 3.

For online lecture, 78.8% of the lecturers selected the Zoom/WebEx/Jitsi/BigBlueButton format, 64.7% the lecture with audio, e.g., with PowerPoint, and 12.9% a live broadcast for the conventional lecture. For the online seminar, 87.7% of the lecturers reported using the Zoom/WebEx/Jitsi/BigBlueButton format, 9.9% a live broadcast of the seminar, and 44.4% the set to audio seminar e.g., with PowerPoint. For online bedside teaching, 87.7% reported using the Zoom/WebEx/Jitsi/BigBlueButton format, 3.2% a live broadcast of the bedside teaching, and 25.8% the bedside teaching set to audio, e.g., with PowerPoint. In addition, the results on the individual instructional formats showed no correlation between gender and the age group queried.

When asked about problems encountered with the internet and the teaching format, 34.7% of the lecturers stated that they had never had problems with the Internet connection, with the general median being 8.0 (IQR: 35.0). In addition, 37% reported never having had problems using the teaching format, with the median here being 7.5 (IQR: 24.0).

Table 2 shows the separate results comparing the analog and digital teaching formats as well as the conversion of the teaching formats before and during the pandemic.

**Table 2.** Detailed results of VAS questions comparing different teaching formats using median and IQR (Interquartile rage) values. Superscript letters indicate significant differences between answer possibilities.

Question No.	Question	Answer possibility	Median	IQR
**20A**	How high do you generally rate the learning success of courses for students?	a) Analog teaching event (Low to high) b) Synchronous formats such as live online or livestreams of lectures, seminars, etc. (Low to high) c) Asynchronous formats like PowerPoint presentations with audio (Low to high)	82.0 ^a^ 61.0 ^b^ 50.0 ^c^	37.5 29.5 41.5
**20B**	In general, how would you rate the opportunities for interaction with students during classes?	a) Analog teaching event (Highly negative to highly positive) b) Synchronous formats such as live online or livestreams of lectures, seminars, etc. (Highly negative to highly positive) c) Asynchronous formats like PowerPoint presentations with audio (Highly negative to highly positive)	100.0 ^a^ 61.0 ^b^ 0.0 ^c^	19.0 38.5 15.0
**21A**	What is your attitude towards online teaching?	a) Before the switch to online-only teaching (Highly negative to highly positive) b) At the present time (Highly negative to highly positive)	37.0 ^a^ 27.0 ^b^	49.0 39.5
**21B**	How much theoretical knowledge do you have about online teaching?	a) Before the switch to online-only teaching (Low to high) b) At the present time (Low to high)	37.0 ^a^ 27.0 ^b^	49.0 39.5
**21C**	How high do you rate your own competence regarding the implementation of an online course?	a) Before the switch to online-only teaching (Low to high) b) At the present time (Low to high)	68.0 ^a^ 69.0 ^a^	35.0 28.0
**21D**	How much of a personal workload do you think your teaching job creates for you?	a) Before the switch to online-only teaching (Low to high) b) At the present time (Low to high)	72.0 ^a^ 65.0 ^a^	27.0 36.0
**22A**	What was/is your time commitment for creating a lecture?	a) Analog lecture (Low to high) b) Digital lecture (Low to high)	71.0 ^a^ 51.0 ^b^	29.0 22.5
**22B**	What was/is your time commitment for delivering a lecture?	a) Analog lecture (Low to high) b) Digital lecture (Low to high)	51.0 ^a^ 80.0 ^b^	24.0 22.5
**22C**	What was/is your time commitment to update an existing lecture?	a) Analog lecture (Low to high) b) Digital lecture (Low to high)	63.0 ^a^ 64.0 ^a^	31.0 26.0
**23A**	What was/is your time commitment for creating a seminar?	a) Analog seminar (Low to high) b) Digital seminar (Low to high)	66.0 ^a^ 55.0 ^b^	27.8 22.8
**23B**	What was/is your time commitment for delivering seminar?	a) Analog seminar (Low to high) b) Digital seminar (Low to high)	50.0 ^a^ 78.0 ^b^	20.5 33.5
**23C**	What was/is your time commitment to update an existing seminar?	a) Analog seminar (Low to high) b) Digital seminar (Low to high)	64.0 ^a^ 58.0 ^a^	28.5 23.5
**24A**	What was/is your time commitment for creating a bedside-teaching?	a) Analog bedside-teaching (Low to high) b) Digital bedside-teaching (Low to high)	50.0 ^a^ 53.0 ^b^	21.5 25.3
**24B**	What was/is your time commitment for delivering bedside-teaching?	a) Analog bedside-teaching (Low to high) b) Digital bedside-teaching (Low to high)	82.0 ^a^ 64.0 ^b^	42.5 37.0

Superscript letters a, b, c indicate significant differences between answer possibilities.

**Figure 4.**
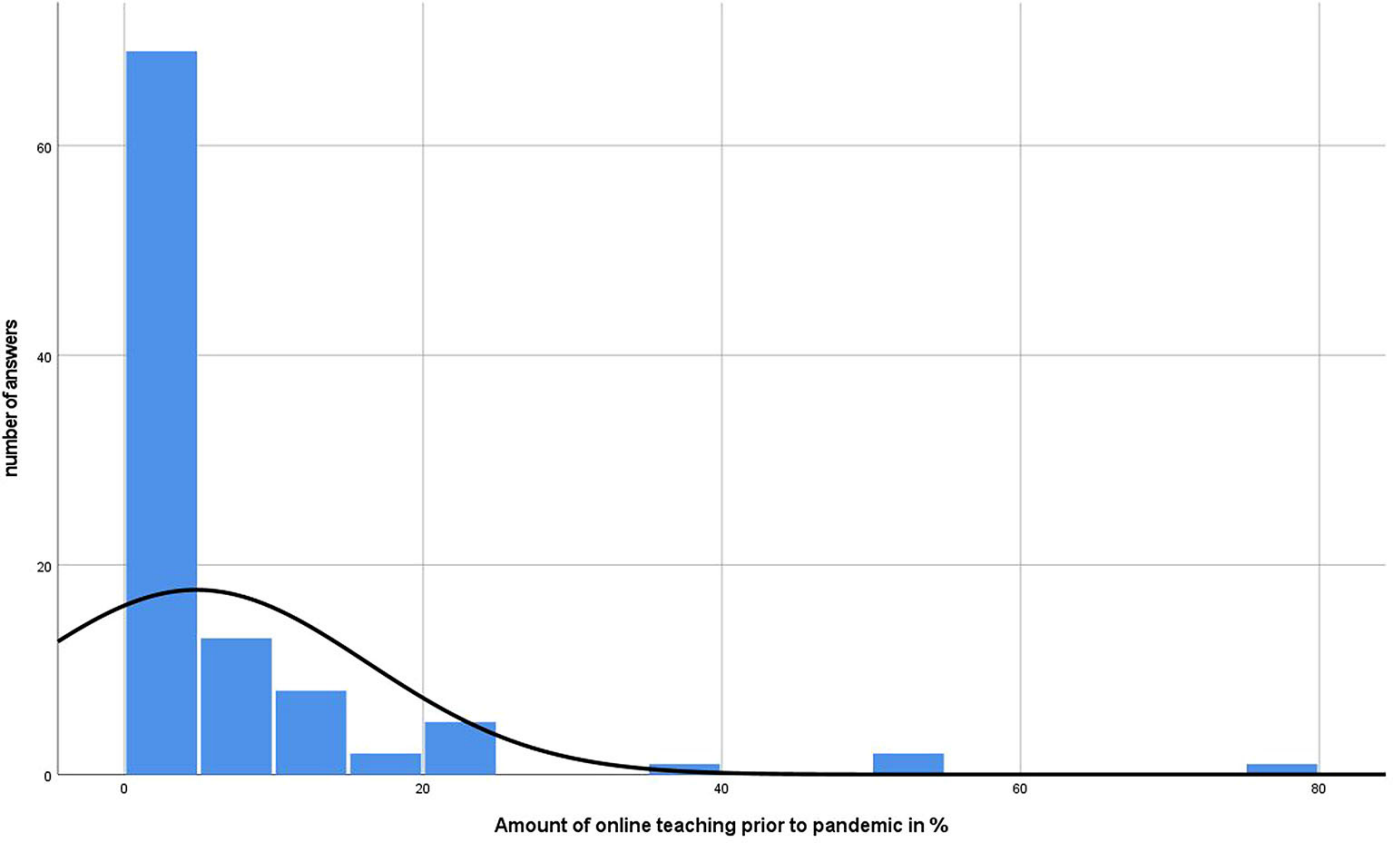
Results of online teaching of participants prior to pandemic in %.

**Figure 5.**
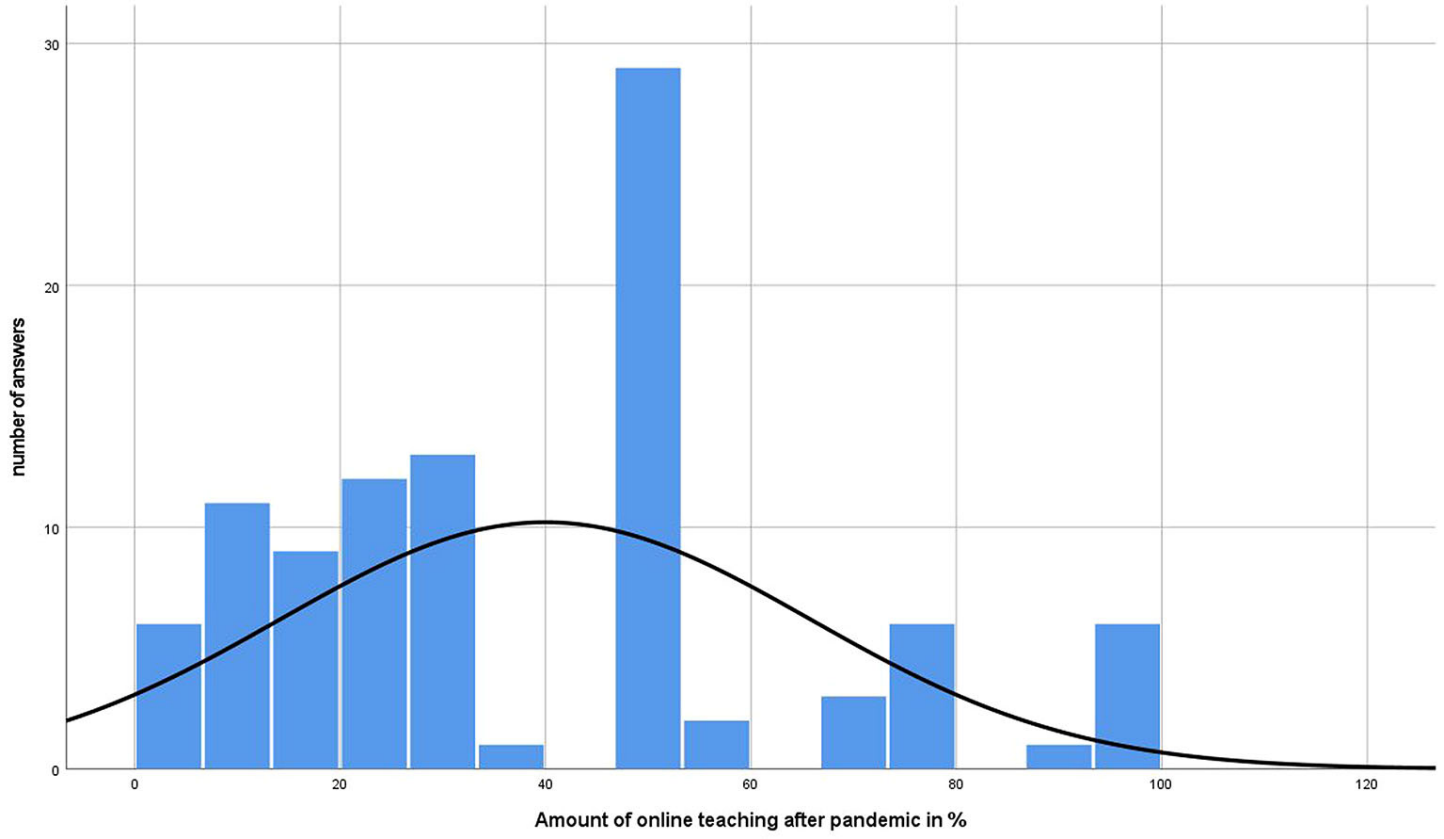
Results of online teaching desire of participants after pandemic in %.

**Figure 6.**
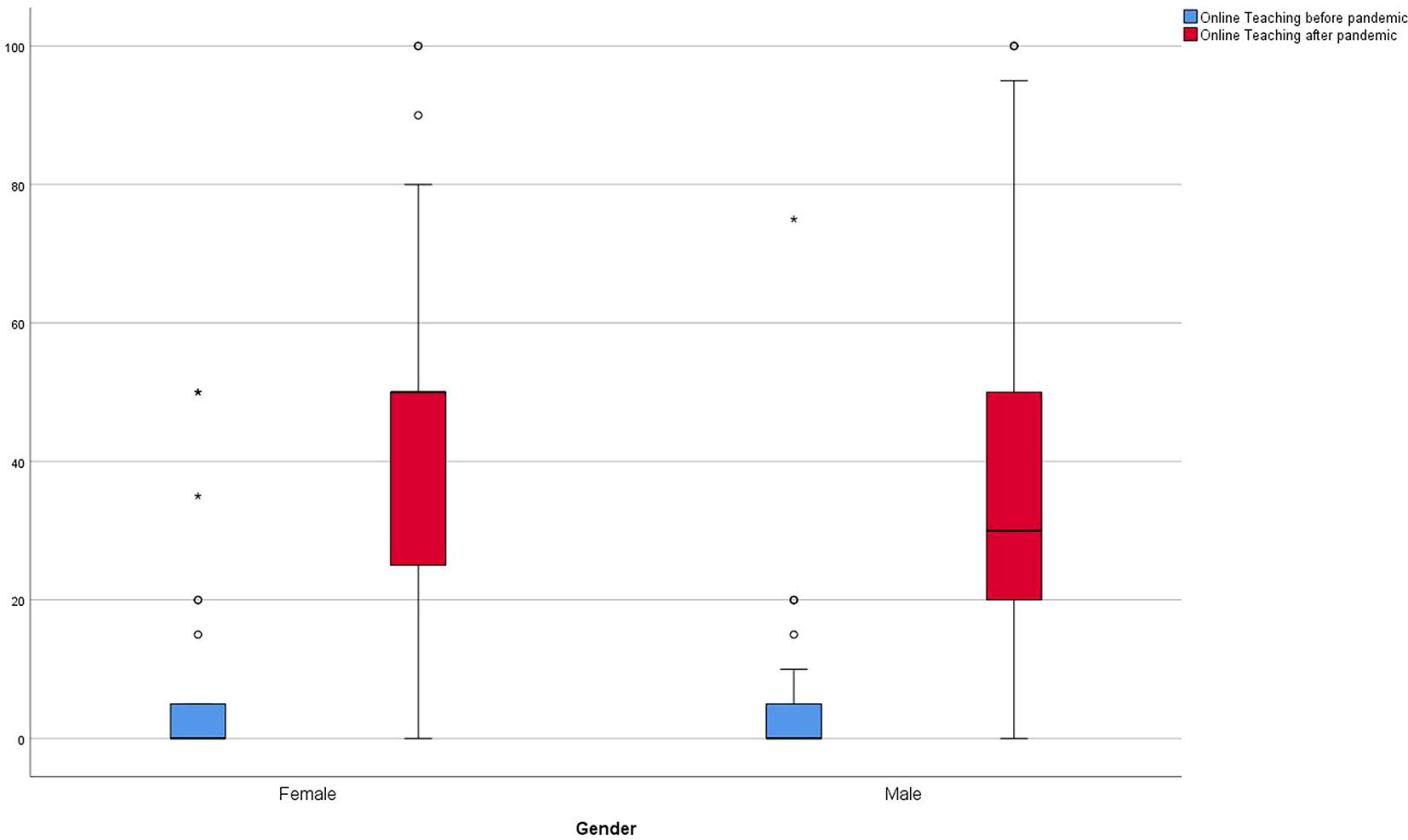
Results of online teaching before and after Covid pandemic of participants in % divided by gender.

Figures 4 and 5 represent the amount of online teaching prior and the desire after the pandemic. In addition, Figure 6 shows the respective results divided by gender.

81.2% of the lecturers are of the opinion that online teaching should in principle be more strongly integrated in dental education in the future; for 18.8%, on the other hand, online teaching should not be more implemented.

## Discussion

The COVID-19 pandemic led to major changes worldwide, especially in social coexistence, as well as work and education. Especially in the field of medical and dental training, which due to their patient-centeredness are related to a high level of physical proximity, fast, alternative solutions had to be sought. An essential way to create infection-preventing distance was the introduction or expansion of digital teaching concepts. Almost all lecturers had to address this often new task. ^23^

The explosive nature of the topic and the concern of almost all lecturers at German universities caused the high number of participants in the present investigation. Although the survey was not based on personalized links or traceable participation, but on voluntary participation, with 101 participating lecturers, a relatively large field of participants could be registered compared to other lecturer surveys. ^18^^,^^24^

The gender and age structure of the present study showed no significant differences between male and female participant numbers between certain age groups. In contrast, the results showed differences in the professional status of the participants. With over 50% of the participants (median 55.4%) the group of the senior physicians, senior physicians; senior consultants or directors are the most frequently represented group. This is not surprising in view of the fact that people in this position often have the predominant teaching performance and teaching responsibility and thus have the highest thematic interest in participating. As a result, most of the study participants were able to state that they had teaching experience of more than 10 years (51.5%).

With 51.5%, more than half of the respondents recorded a share of 25% and 50% in teaching, referred to their entire professional activity. 94.1% of those questioned also stated that more than 25% of their work was done outside of teaching. Clinical dental work beyond of teaching usually does not take place at home. Therefore, many lecturers are tied to one specific location. So at least 94.1% cannot fully exploit the advantages of digital teaching concepts. ^21^ This restriction could be assumed to be the cause of the fact that only 13.9% of the respondents stated that they teach from home, while 83.1% of the study participants locate their teaching activities in the university (67.3% from the university’s own office and 15.8% from classrooms such as lecture halls or conference rooms). In this regard other authors report similar results. Schlenz *et al.,* ^18^ identified share of 60.0% of the lecturers who gave lectures from the university’s own office, while 2.9% used specially equipped conference rooms.

Ebner *et al.,* ^25^ described a worldwide hype of digital teaching at universities, triggered by the pandemic. How much this trend has changed in the field of dental teaching was shown by the question of experience with online teaching. Although the acceptance, the usefulness and the effectiveness of digital learning formats have been described for decades, ^10^^,^^12^^,^^14^^–^^17^^,^^26^^–^^28^ only 24.8% of respondents said that they had offered digital teaching prior to pandemic. In addition, 64.4% of the participating lecturers had no previous experience. These results were in line with previous investigations. ^18^ After the outbreak of the pandemic, more than 80% of the lecturers said they were doing online lectures (86.1%), online seminars (81.2%), and/or online bedside teaching (33.7%), which represented a sharp increase.

This rapid development in the implementation of digital content in the dental curriculum also harbors certain dangers. In spring 2020, COVID-19 caused considerable restrictions in dental teaching. ^23^ The rapid changes in university teaching that became necessary as a result hit the universities and lecturers suddenly. As the present study shows, only 25.7% of the respondents were able to state that they had taken part in corresponding advanced training events on digital teaching by the time the pandemic broke out, and 36.6% of the respondents had dealt with the topic at least in self-study.

It can be assumed that, not least because of the lack of experience with digital teaching concepts until then, the selection of the teaching format was specified by the university for 62.4% of the participants. Against the background of countless digital possibilities in the field of teaching, ^20^ this appears to be a sensible measure to create clear university structures. In addition, the license agreements represented a certain university restriction and focus on individual suppliers. Since a large number of students must be able to attend synchronous lectures, seminars, and bedside teaching in digital formats, licenses are often unavoidable.

Although authors who examined the acceptance of digital teaching concepts on the part of students reported very high acceptance values, combined with the demand for more online teaching, ^2^ this development was not clearly reflected on the part of the lecturers.

With 88.1% of the respondents, the vast majority indicated that classroom teaching was the preferred form of teaching, despite having had experience with digital teaching in the meantime. The reasons for this were certainly multilayered. For many lecturers, one reason could be the sudden need for digital offers combined with a lack of previous experience. This situation possibly led to excessive demands, at least temporarily. It should not be forgotten that digital teaching concepts also has disadvantages that are often described, such as eye fatigue from working on the screen and thus a decrease in receptivity or a lack of motivation. ^4^^,^^29^ Another decisive disadvantage of digital teaching concepts is the reduced interaction both between students and between students and lecturers. ^30^ The selection of the teaching format suggests that this point is not insignificant on the lecturer side. Although formats in which discussed slides are used and which can be called up repeatedly, if necessary, with slight modifications, can be ‘recycled’ again and again, the preferred teaching formats, regardless of the type of course, were the “live” formats such as Zoom, WebEx, Jitsi, or BigBlueButton. These formats allow far more direct communication.

At the same time, the participants also evaluated the learning success of analog teaching as the greatest. On a scale from 0-100, the participants gave the analog lessons a median value of 82.0 for learning success, while digital, synchronous formats were rated significantly worse with a median value of 61.0. The participants rated the digital asynchronous teaching significantly worse.

The participants evaluated the differences in the interaction options with similar clarity, but even more clearly. Corresponding to the assessment of the learning success, the respondents see the greatest opportunities for interaction in the area of analog teaching (median 100.0). The opportunities for interaction with synchronous digital teaching (median 61.0) are seen as significantly worse. In asynchronous digital teaching, the lecturers see almost no opportunity for interaction (median 0.0).

Although a majority of the lecturers still prefer to use analog teaching and consider this to be more productive, the attitude towards online teaching has changed positively. While the respondents rated their attitudes towards online teaching on a scale of 0-100 with a median of 24.0, this improved during the pandemic to a median value of 50.0.

At the same time, however, in the relatively short period of the pandemic, the lecturers neither had the perceived knowledge of digital teaching concepts (before median 60.0 after 77.0) nor the perceived competence in the implementation of digital content (before median 68.0, After 69.0) significantly improved.

Surprisingly, the surveyed lecturers did not perceive any difference in the workload of their teaching activity between the workload before and during the pandemic. Although there was a tendency to describe additional effort before the pandemic, it was not statistically significant. When asked about the effort required to create individual teaching formats, a mixed picture emerges. While a significant additional effort is seen in the creation of lectures with digital formats (analog median 50.0, digital median 73.0), there is no significant difference between the creation of digital and analog formats when creating seminars and bedside teaching. This result could possibly be explained with the workflow of the various creation modes. While bedside teaching and seminars focus on working out certain issues in small groups, the lecturer takes the active part in lectures, while the students usually only receive the information. ^31^ The second one is therefore better suited to be reproduced multiple times in asynchronous form. However, due to the storage of audio files, this requires more creation effort than digital lectures. The seminars or bedside-teaching, which are mostly offered as synchronous formats, differ little in terms of creation effort from their analog form.

When presenting the teaching units, on the other hand, the respondents see a significant additional effort both in the area of the lectures and in the area of the seminars. Only with bedside teaching a significant additional effort is seen in the analog form of learning.

When updating existing teaching units, however, this difference was small, so that no significant difference could be analyzed either in lectures (analog median 63.0, digital median 64.0) or in seminars (analog median 64.0, digital median 58.0).

Basically, the results clearly show how university dental teaching has changed since the pandemic. As already described in previous studies, the surveyed lecturers would like to continue online teaching after the pandemic. ^18^ While the respondents in this study estimated the median proportion of their online teaching before the pandemic to be 4.8% (SD ± 11.44), they hope for a proportion of 40% (SD ± 25.8) in the future. The results suggest that female lecturers in particular are in favor of implementing more online teaching (male 30 %, female 50%).

Due to its high proportion of crucial practical skills, dental training is not suitable for being fully taught in distance learning. However, the results show that there is a high level of willingness on the part of the lecturers to continue to design at least the theoretical part of the training digitally. At the same time, the participating lecturers point out the weaknesses of digital teaching in the area of interaction, which should be reduced as much as possible by choosing the appropriate format.

The present study had some limitations, on the one hand, in the composition of the field of participants and, on the other hand, in the circumstances in which the switch to digital teaching took place.

Since participation in this study was voluntary, a field of participants that was unevenly large between the various universities emerged. In order to take this fact into account, the present results must be viewed taking into account the weighting shown in Figure 2. Voluntary participation also means that only the more motivated employees pre-selectively declared themselves study participants. As we had no information about how many employees of the other universities received the questionnaire and how many did not participate, it was not possible to determine a drop-out rate.

Another limitation is the current situation under which the switch to digital teaching offers was mostly not voluntary. Here it would be advisable to collect the results again in further studies with a suitable interval. This would allow a realistic assessment of the attitudes towards digital teaching concepts on the part of the lecturers.

In summary, it can be stated that although the study participants offer significantly more digital teaching in times of the pandemic and have significantly positively changed their attitude towards online teaching, the learning success of analog teaching is rated highest. In the case of lectures in particular, the participating lecturers see a considerable amount of additional work involved in creating digital files. In general, the teachers at German universities are positive about the increased use of digital teaching and hope to be able to offer a far higher proportion of the teaching units digitally in the future.

## Conclusion

Within the limitations of the present cross-sectional survey-based investigation, the following conclusions could be drawn:

1. Faculty members positively changed their attitude towards online teaching formats during the COVID-19 pandemic.
2. Lecturers rated the learning success with analog face-to-face teaching formats the highest.
3. Lecturers evaluated an additional effort in creating digital lectures.
4. More online teaching would be preferred by lecturers in the future.

## Data availability

### Underlying data

Open Science Framework. Dental education during the pandemic – cross-sectional lecturer-side evaluation for the use of digital teaching concepts. https://doi.org/10.17605/OSF.IO/QD9KT. ^32^

The project contains the following underlying data:

• Excel_File_b.xlsx. (raw underlying data of anonymized questionnaire responses).
• Excel_File_b.csv (raw underlying data of anonymized questionnaire responses).

### Extended data

The project contains the following extended data:

• Questionnaire unvalidated. (Original unvalidated questionnaire in English).
• Questionnaire unvalidated German. (Original unvalidated questionnaire in German).
• Questionnaire Final validated (Final validated English questionnaire used in this study).
• Questionnaire Final validated German (Final validated German questionnaire used in this study).

Data are available under the terms of the Creative Commons Zero “No rights reserved” data waiver (CC0 1.0 Public domain dedication).
