# Supplementary material for: Dental education during the COVID-19 pandemic in Germany – cross-sectional  lecturer-side evaluation for the application of digital teaching concepts
Source: F1000Res. 2022 Nov 9;11:767. Originally published 2022 Jul 11. [Version 2] doi: 10.12688/f1000research.122274.2 (PMC9554493; doi:10.12688/f1000research.122274.2)
Supplement: Peer review history

# Peer review: Reviewer response for version 2

**DOI:** 10.5256/f1000research.140113.r155404

**Published:** 2022-11-10

**Authors:** Maximiliane Amelie Schlenz

**Affiliations:** 1 Department of Prosthodontics, Justus-Liebig-Universität Giessen, Giessen, Germany

The authors addressed all concerns well. I suggest publishing the manuscript. Thank you very much for this valuable data!

Is the work clearly and accurately presented and does it cite the current literature?

Yes

If applicable, is the statistical analysis and its interpretation appropriate?

Yes

Are all the source data underlying the results available to ensure full reproducibility?

Yes

Is the study design appropriate and is the work technically sound?

Yes

Are the conclusions drawn adequately supported by the results?

Yes

Are sufficient details of methods and analysis provided to allow replication by others?

Yes

Reviewer Expertise:

digital dentistry, intraoral scanners, restorative dentistry, clinical dentistry, dental education, dental materials, implant prosthetic, removable and fixed prothodontics, gerodontology

I confirm that I have read this submission and believe that I have an appropriate level of expertise to confirm that it is of an acceptable scientific standard.

# Peer review: Reviewer response for version 2

**DOI:** 10.5256/f1000research.140113.r155403

**Published:** 2022-11-10

**Authors:** Mohammed Gamal

**Affiliations:** 1 Department of Pharmaceutical Chemistry, College of Pharmacy, Al-Jouf University, Aljouf, Saudi Arabia

The authors replied for all reviewers' comments in the first round in a very professional way. The paper could be published now in the current form. I appreciate their efforts.

Is the work clearly and accurately presented and does it cite the current literature?

Partly

If applicable, is the statistical analysis and its interpretation appropriate?

Yes

Are all the source data underlying the results available to ensure full reproducibility?

Yes

Is the study design appropriate and is the work technically sound?

Yes

Are the conclusions drawn adequately supported by the results?

Yes

Are sufficient details of methods and analysis provided to allow replication by others?

Yes

Reviewer Expertise:

pharmaceutical chemistry, green chemistry , instrumental analysis, medical survey research , clinical sciences

I confirm that I have read this submission and believe that I have an appropriate level of expertise to confirm that it is of an acceptable scientific standard.

# Peer review: Reviewer response for version 1

**DOI:** 10.5256/f1000research.134243.r151554

**Published:** 2022-10-11

**Authors:** Jens Christoph Türp

**Affiliations:** 1 University Center for Dental Medicine Basel UZB, Department of Oral Health, University of Basel, Basel, Switzerland

The article by the five authors addresses a timely topic: teaching in times of the Covid 19 epidemic. Specifically, they examined how digital teaching in dentistry (here: prosthodontics) has changed as a result of the pandemic ‒ and how faculty have responded to the unexpected challenges.

 I will share my impressions, comments, and suggestions below. Before doing so, I would like to emphasize that I agree with all the suggestions made by the reviewer Maximiliane Amelie Schlenz (University of Giessen). Therefore, I will not address the manuscript-related points mentioned by her in my review.

 In the following, I will present my points in chronological order according to the sequence of the individual parts of the manuscript.

**ABSTRACT**

 (1) Background: the authors should better visually emphasize the two aims of their study by labeling them "(a)" and "(b)": "The aim of this investigation was to evaluate (a) the attitudes of [...] and (b) the effort creating digital lectures."

 (2) Results: The precise numbers (24.8%, 64.4%, 86.1%, 81.2%, 33.7%, etc.) given to one decimal place suggest a mathematical accuracy, which in reality ‒ not least because of the methodology (see later) ‒ is rather due to chance. It would therefore be more advantageous to paraphrase the calculated values linguistically instead, e.g., by writing "a quarter", "far more than half", "the vast majority", "about a third", etc.

 (3) Results: In contrast to the German language, sentences in English must not begin with Arabic numerals ("88.1% see face-to-face teaching"). Instead, in such cases, the numbers are written out. In this specific case, however (see point 2 above), a paraphrase like "Four out of five lecturers see ..." would be the better linguistic choice.

 (4) Results: Results are presented by the authors only for the first objective, but none for the second aim (effort/workload).

 (5) Results: The first two sentences of the results provide information for which no aim was provided in "Background". Therefore, one could add another ‒ first ‒ aim there: "[...] was to evaluate the changes in the percentage of digital teaching."

 (6) Conclusions: they are relatively trivial. The results provide possibilities for a more attractive conclusion.

**INTRODUCTION**

 (1) 2nd paragraph, end of the first sentence: "still has deficiencies". The reader would certainly like to know which deficiencies are involved.

 (2) 3rd paragraph, second sentence: "blended learning". A brief explanation of what is meant by this term would be helpful since it cannot be assumed that all readers of the article are familiar with it.

 (3) 4th paragraph, first sentence: (a) "described by the students". Which students are meant and to what does this statement refer? (b) "Investigations" can no more be "found" than studies can be read. Investigations/studies are planned, funded, conducted, and completed, but never found or read. What is "found" are articles in a literature search, and what is "read" are articles about a study.

 (4) Paragraphs 4 and 5: Both begin with "Although". This can easily be improved.

**METHODS**

 (1) 2 ^nd^ paragraph (Study design): This paragraph belongs in the introduction.

 (2) 3 ^rd^ paragraph (Participant recruitment): "All German dental schools". The reader would certainly like to know how many dental schools there are in Germany.

 (3) 3 ^rd^ paragraph (Participant recruitment): Of great importance is an indication of whether there was a maximum number of study participants per site ‒ and if not, why not. If you look at Figure 2, you can see why this question is important (possibility of bias in the results). The authors should also mention whether external lecturers, i.e., lecturers not permanently employed at the dental clinic (e.g., lecturers working in private practice [external senior physicians/dentists; *Privatdozenten*; adjunct professors]) were also allowed to participate in the study.

 (4) 4 ^th^ paragraph (Questionnaire development): The explanations are partly incomprehensible, especially the third last sentence ("After validation, a short cut was created from ..."). More detailed explanations are required here. The aim of a good methodology section should always be to enable the reader, at least theoretically, to repeat the study without asking the authors. For this purpose, the authors must disclose from the beginning everything that is necessary to understand the applied methodology.

 (5) 5th paragraph (Data analysis): "The questionnaires were examined with ...". By whom were they examined/evaluated?

 (6) Figure 1: The scale should have two anchor points labeled "0" and "100".

**RESULTS**

 (1) Fig. 2: It is clear from this figure that the results obtained in this study are very unlikely to be representative of dental prosthodontic teaching in Germany, but rather a biased sample. Of the 30 (?) dental universities in Germany, only 18 are represented in this study. These 18 are weighted differently. Not unexpectedly, study participants from the University of Munich make up the majority with about one-fifth of the total number. Interestingly, no one from the University of Cologne, where one of the authors is from, participated. This bias does not devalue the study, but it does diminish its general validity. This drawback should be highlighted more in the discussion than the authors do in a brief section toward the end of the discussion.

 (2) In the text, as already mentioned above in the "Abstract" section, pseudo-exact numerical values should be avoided in favor of rounding or linguistic descriptions. What on earth are "34.7% of the lecturers" in view of 84 participants ("101 lecturers" minus "17 lecturers with incomplete questionnaires"; see Results, 1st par.)?

 (3) Paragraph 8: "lecture with audio, e.g., with PowerPoint". PowerPoint is primarily visual, not audio. 

 (4) Paragraph 9: What exactly do the "general medians" indicate? What do they refer to? How do the numerical values come about?

 (5) Table 2: The given values of the medians and IQR should be at the level of the respective answer options a), b) and c).

**DISCUSSION**

 (1) A good discussion always starts with the highlight of the study results (" *Where is the meat?*"), but not with general statements like "The COVID-19 pandemic led to major changes worldwide, ..." - everyone knows that.

 (2) The discussion is unnecessarily long. The probability that an article will be read increases if it is kept short.

 (3) What has been pointed out before also applies to the discussion: Avoid giving exact numbers from the study.

 (4) The authors compare the two teaching formats analogue (lecture hall; seminar room) versus digital (computer). Possibly, however, another aspect plays a role: the lecturer. After all, until the beginning of the pandemic, holding online lectures was never an issue for her/him. Now she/he is forced to speak into a microphone in an unfamiliar environment (her/his office, her/his home) and an unfamiliar format, sitting in front of his computer. You could say: instead of a familiar theatre performance, she/he is suddenly forced to deliver a radio play, a format completely unfamiliar to her/him. This change is not easy for every lecturer. Consequently, some lecturers deliver a presentation that is below the standards they have been used to in the lecture hall; in addition, they have to deal with technical problems, speak in a monotone voice, etc. In such cases, however, the differences in the evaluations concern less the teaching format, but rather the performance of the lecturer. This aspect should be examined more closely in the discussion.

 (5) Paragraph 14: "bedside-teaching". Do the authors mean "chairside teaching"?

 (6) Paragraphs 15 and 16: Both start with "When".

**CONCLUSION**

 (1) What does the following sentence mean: "Lecturers evaluated an additional effort in creating digital lectures"?

**REFERENCES**

 (1) Ref. 29 and 30: The titles of the papers are given in English. However, the articles have been published in German. The corresponding original German titles are:

 29: Organizational Embedding of E-Learning at German Universities. Institute for Information Management Bremen

 30: Change Management in Higher Education: The Sustainable Implementation of e-Learning Innovations.

 A disregard of the original title and replacement of the same by an own translation (which in the case of reference 30 is inaccurate, since it should read: Change Management in University Teaching: The Sustainable Implementation of e-Learning Innovations) is not permitted without a corresponding note.

 (2) The following literature should be included ^1^^,^^2^^,^^3^

**GENERAL REMARKS**

 (1) The text needs linguistic (English) improvement.

 (2) Sentences in scientific English are shorter than in German. Therefore, two shorter sentences are better than one long one.

Is the work clearly and accurately presented and does it cite the current literature?

Partly

If applicable, is the statistical analysis and its interpretation appropriate?

I cannot comment. A qualified statistician is required.

Are all the source data underlying the results available to ensure full reproducibility?

Partly

Is the study design appropriate and is the work technically sound?

Partly

Are the conclusions drawn adequately supported by the results?

Yes

Are sufficient details of methods and analysis provided to allow replication by others?

Partly

Reviewer Expertise:

Evidence-based dentistry, orofacial pain, temporomandibular disorders, occlusion

I confirm that I have read this submission and believe that I have an appropriate level of expertise to confirm that it is of an acceptable scientific standard, however I have significant reservations, as outlined above.

## References

[1] : Student perspective of classroom and distance learning during COVID-19 pandemic in the undergraduate dental study program Universitas Indonesia. *BMC Med Educ* .2020;20(1) : 10.1186/s12909-020-02312-0 392 10.1186/s12909-020-02312-0 33121488PMC7594975

[2] : Dental education during the pandemic: Cross-sectional evaluation of four different teaching concepts. *J Dent Educ* .2021;85(10) : 10.1002/jdd.12653 1574-1587 10.1002/jdd.12653 34046898PMC8242849

[3] : When e-learning takes centre stage amid COVID-19: Dental educators' perspectives and their future impacts. *Eur J Dent Educ* .2022;26(3) : 10.1111/eje.12727 506-515 10.1111/eje.12727 34813667PMC9011897

# Peer review: Reviewer response for version 1

**DOI:** 10.5256/f1000research.134243.r151553

**Published:** 2022-09-27

**Authors:** Mohammed Gamal

**Affiliations:** 1 Department of Pharmaceutical Chemistry, College of Pharmacy, Al-Jouf University, Aljouf, Saudi Arabia

The article is very interesting. It is very well written in a very clear language. It worth publication after minor revisions.

 1. Title should be modified to be dental education in Germany to reflect the geographical location for the study.

 2. The following article should be cited ^1^^,^^2^^,^^3^^,^^4^

 3. Comparisons of dental education studies from lecturers points of view from different developed countries e.g. UK, USA, during pandemic should be stated and illustrated in discussions.

 4. Introduction should be ended with the main aim of the research paper. Therefore, I recommend reorganization of the last paragraph in introduction to be above aim statements “The hypothesis states that on the lecturer side 1. There is no difference between the various digital teaching concepts in terms of workload and effort, and 2. There is no increase in workload and effort when switching to digital teaching concepts.”

 5. Date of ethical statement should be provided.

 6. In study design [All dental schools, like all other universities] add in Germany.

 7. In table 2 , results should be aligned with choices i.e. in the same level on the same line.

 8. Future research plans and study limitations should be provided.

Is the work clearly and accurately presented and does it cite the current literature?

Partly

If applicable, is the statistical analysis and its interpretation appropriate?

Yes

Are all the source data underlying the results available to ensure full reproducibility?

Yes

Is the study design appropriate and is the work technically sound?

Yes

Are the conclusions drawn adequately supported by the results?

Yes

Are sufficient details of methods and analysis provided to allow replication by others?

Yes

Reviewer Expertise:

pharmaceutical chemistry, green chemistry , instrumental analysis, medical survey research , clinical sciences

I confirm that I have read this submission and believe that I have an appropriate level of expertise to confirm that it is of an acceptable scientific standard.

## References

[1] : Dental education during the pandemic: Cross-sectional evaluation of four different teaching concepts. *J Dent Educ* .2021;85(10) : 10.1002/jdd.12653 1574-1587 10.1002/jdd.12653 34046898PMC8242849

[2] : Satisfaction with online teaching of medical statistics during the COVID-19 pandemic: A survey by the Education Committee of the Italian Society of Medical Statistics and Clinical Epidemiology. *Teach Stat* .2021;43(3) : 10.1111/test.12286 129-139 10.1111/test.12286 34548710PMC8447082

[3] : Faculty perceptions on online education in a school of pharmacy during the COVID-19 pandemic. *Pharmacy Education* .2022;22(1) : 10.46542/pe.2022.221.450457 450-457 10.46542/pe.2022.221.450457

[4] : Online dental teaching practices during the COVID-19 pandemic: a cross-sectional online survey from China. *BMC Oral Health* .2021;21(1) : 10.1186/s12903-021-01547-7 10.1186/s12903-021-01547-7 33845828PMC8040365

# Response

**Published:** 2022-10-20

**Authors:** Anja Liebermann

**Affiliations:** Medical Center of the Ludwig-Maximilians-University, Germany

Dear Mr Gamal,

 Dear Ms Schlenz,

 Thank you for taking the time to edit our article.

 We tried to consider your comments to the best of our knowledge.

 We changed the title of the manuscript, as suggested by Mr. Gamal and Ms. Schlenz, and incorporated the references described by Mr. Gamal in the Introduction chapter.

 Unfortunately, we could not find any comparable studies in dentistry that examine the change in teaching from the lecturer's perspective during the pandemic. We will pursue the literature on this topic and supplement the article if necessary.

 In the Introduction chapter, we took up Mr. Gamal's suggestion and reorganized the last paragraph.

 Furthermore, we have included the date of the ethical statement in the text and added in the study design that the universities in question are all located in Germany.

 In the discussion, we described in more detail the limitations of this study.

 The mentioned formatting in Table 2 presented us with challenges. While the criticism for the PDF format is valid, in the online version of the article the results are aligned with choices in the same line. We provided Table 2 with appropriate values (Low:0, high:100)..The same applies to the font size of the illustrations mentioned by Ms. Schlenz. Again, the criticism only applies to the PDF version of the article, but not to the online version. Appropriate formatting of f1000reaserch may help here.

 We have gratefully complied with the notification of the uniform formulations.

 We also briefly described the established German presence concept and expanded the hypotheses to include this aspect.

 Also, we added the requested period to the article.

 In order to enable better interpretability of the data, we have provided the scroll bar with the appropriate values ​​(0 to 100).

 We hope to have taken up your suggestions in the desired way and remain with kind regards.

# Peer review: Reviewer response for version 1

**DOI:** 10.5256/f1000research.134243.r143883

**Published:** 2022-07-26

**Authors:** Maximiliane Amelie Schlenz

**Affiliations:** 1 Department of Prosthodontics, Justus-Liebig-Universität Giessen, Giessen, Germany

Thank you for the opportunity to review the manuscript “Dental education during the pandemic – cross-sectional lecturer-side evaluation for the use of digital teaching concepts”.

 The general approach to investigating the lecturers' perception towards digital teaching across all German Dental Schools is of high interest, not only limited to the topic of teaching during the COVID-19 pandemic, but rather for new digital teaching opportunities related to the new medical licensure act for dentistry in Germany. Furthermore, this study provides valuable information for lecturers around the world that like to implement or improve digital teaching in dental education.

 I have to emphasize the elaborate Discussion section and clear subsumption of the results to the available literature. 

 Answers to F1000 questions according to guidelines for reviewing:

- The study design is appropriate and the work have an academic merit.
- The work is clearly and accurately presented and the relevant current literature is cited.
- The materials and methods section contains sufficient details to replica the study by others.
- As far as I can see, the source data underlying the results are available.
- The statistical analysis is appropriate and well interpreted.
- The conclusions drawn are adequately supported by the results of the study.

From my point of view, the manuscript is general acceptable for publication. However, I suggest to address the following small points:

1. Please, revise the manuscript regarding a consistent wording (e.g. analog or conventional, classroom teaching or event). Otherwise, the readers might get confused.
2. I suggest to revise the title: “Dental education during the COVID-19 pandemic – cross-sectional lecturer-side evaluation for the application of digital teaching concepts”.
3. Please, extend the null hypothesis regarding the aspects “handling/attitude” (Questions 10-21C) towards digital teaching. These valuable aspects are not addressed in the null hypothesis yet, because only workload and effort are considered.
4. I suggest to add a paragraph describing the established German face-to-face teaching concept to the introduction section, because in other countries digital concepts might be already implemented before COVID-19 pandemic.
5. Add the investigated aspects “handling/attitude” (Questions 10-21C) to the Introduction section and highlight more the key aspect of your questionnaire survey not investigating just one single dental school, but rather including all locations in Germany.
6. Please, add the period that you asked the lecturers for. I guess you were interested in the period March 2020 to January 2021?
7. For better clearance, add the measured data (0 to 100) to the scroll bar in Figure 1. Otherwise, it is a little bit difficult to interpret data of Table 2. In addition, I suggest explaining the analysis of the scroll bar in the legend of Table 2 and design the column “Answer possibility” clearer.
8. Please, increase the font of Figure 2-6.

I suggest to approve the manuscript with reservations.

 Good luck and keep well!

Is the work clearly and accurately presented and does it cite the current literature?

Yes

If applicable, is the statistical analysis and its interpretation appropriate?

Yes

Are all the source data underlying the results available to ensure full reproducibility?

Yes

Is the study design appropriate and is the work technically sound?

Yes

Are the conclusions drawn adequately supported by the results?

Yes

Are sufficient details of methods and analysis provided to allow replication by others?

Yes

Reviewer Expertise:

digital dentistry, intraoral scanners, restorative dentistry, clinical dentistry, dental education, dental materials, implant prosthetic, removable and fixed prothodontics, gerodontology

I confirm that I have read this submission and believe that I have an appropriate level of expertise to confirm that it is of an acceptable scientific standard, however I have significant reservations, as outlined above.

# Response

**Published:** 2022-10-20

**Authors:** Anja Liebermann

**Affiliations:** Medical Center of the Ludwig-Maximilians-University, Germany

Dear Mr Gamal,

 Dear Ms Schlenz,

 Thank you for taking the time to edit our article.

 We tried to consider your comments to the best of our knowledge.

 We changed the title of the manuscript, as suggested by Mr. Gamal and Ms. Schlenz, and incorporated the references described by Mr. Gamal in the Introduction chapter.

 Unfortunately, we could not find any comparable studies in dentistry that examine the change in teaching from the lecturer's perspective during the pandemic. We will pursue the literature on this topic and supplement the article if necessary.

 In the Introduction chapter, we took up Mr. Gamal's suggestion and reorganized the last paragraph.

 Furthermore, we have included the date of the ethical statement in the text and added in the study design that the universities in question are all located in Germany.

 In the discussion, we described in more detail the limitations of this study.

 The mentioned formatting in Table 2 presented us with challenges. While the criticism for the PDF format is valid, in the online version of the article the results are aligned with choices in the same line. We provided Table 2 with appropriate values (Low:0, high:100). The same applies to the font size of the illustrations mentioned by Ms. Schlenz. Again, the criticism only applies to the PDF version of the article, but not to the online version. Appropriate formatting of f1000reaserch may help here.

 We have gratefully complied with the notification of the uniform formulations.

 We also briefly described the established German presence concept and expanded the hypotheses to include this aspect.

 Also we added the requested period to the article.

 In order to enable better interpretability of the data, we have provided the scroll bar with the appropriate values ​​(0 to 100).

 We hope to have taken up your suggestions in the desired way and remain with kind regards.
